# Knowledge, and attitude, and decisional conflict regarding biologics among patients with inflammatory bowel disease: a cross-sectional study

**DOI:** 10.3389/fmed.2025.1604851

**Published:** 2025-10-09

**Authors:** Qianqian Li, Chenmei Xia, Chunxiao Chen, Yi Jiang, Lingxiao Jin, Jianmei Zhang, Linfeng Wu, Li Jiang, Xia Chen

**Affiliations:** ^1^Department of Gastroenterology, The First People’s Hospital of Wenling (Taizhou University Affiliated Wenling Hospital), School of Medicine, Taizhou University, Taizhou, China; ^2^Department of Gastroenterology, The First Affiliated Hospital, Zhejiang University School of Medicine, Hangzhou, China; ^3^Department of Gastroenterology, The Second Affiliated Hospital of Wenzhou Medical University, Wenzhou, China; ^4^Department of Gastroenterology, Lishui People’s Hospital, Lishui, China; ^5^Department of Anesthesiology, Taizhou Central Hospital, Taizhou, China

**Keywords:** inflammatory bowel diseases, biological therapy, knowledge, attitude to health, decision making

## Abstract

**Objective:**

This study aimed to investigate the knowledge, attitude, and decisional conflict regarding biologic treatments among patients with inflammatory bowel disease (IBD) in China.

**Methods:**

This cross-sectional study included IBD patients recruited at the authors’ hospital between July 2023 and January 2024. Data were collected using a self-administered questionnaire covering demographic characteristics, knowledge, attitude, and decisional conflict (measured by the Decisional Conflict Scale).

**Results:**

A total of 405 IBD patients participated, with 45.9% aged 30–49 years and 64% male. The average knowledge score was 6.03 ± 2.98 (possible range: 0–11), and the average attitude score was 24.36 ± 3.32 (possible range: 8–40). Among them, 60 (14.8%) patients exhibited significant decisional conflict. Spearman correlation analysis revealed a positive association between knowledge and attitude scores (*r* = 0.554, *p* < 0.001). Multivariate analysis revealed that being ≥ 50 years old, having ulcerative colitis, never having used biologics, and experiencing significant decisional conflict were associated with lower knowledge scores. Higher knowledge scores, a monthly income between 10,000–20,000 RMB, never having used biologics, and significant decisional conflict were associated with lower attitude.

**Conclusion:**

Older age, ulcerative colitis, lack of prior biologic use, and decisional conflict were associated with lower knowledge and lower attitude toward biologics among IBD patients. Targeted educational interventions may help reduce decisional conflict and improve patients’ attitudes toward biologic therapy.

## Introduction

Inflammatory bowel disease (IBD) represents a complex group of chronic gastrointestinal conditions, prominently including Crohn’s disease (CD) and ulcerative colitis (UC). These disorders are characterized by an unpredictable course of relapse and remission, profoundly affecting the quality of life of patients (1). The epidemiological landscape of IBD is marked by a growing prevalence worldwide due to chronicity with a lack of cure, the young age of onset, and low mortality, which places a substantial burden on patients and healthcare systems. IBD is currently known to affect over 6 million people worldwide (2, 3). The etiology of IBD is multifaceted and involves environmental, genetic, and immunological factors, making its management particularly challenging (4, 5).

Biologic therapies have revolutionized the treatment landscape for IBD by specifically targeting immune pathways, such as the tumor necrosis factor, which plays a pivotal role in the inflammatory process (6, 7) The adoption of biologics has been increasing due to their efficacy in reducing inflammation, maintaining long-term remission, and decreasing the necessity for surgical interventions. This therapeutic advancement has significantly improved the management outcomes and the overall quality of life for patients with IBD (8, 9). However, in real-world clinical practice, the uptake of biologics depends not only on medical indications but also on patients’ understanding, perception, and acceptance of these treatments, which can vary widely across populations and healthcare systems.

Although biologics offer numerous advantages, some have been associated with severe side effects, such as an increased risk of lymphoma, serious infections, and immunological reactions. The diverse and complex risk–benefit profiles of various biologics, coupled with the growing range of treatment options, complicate the decision-making process for patients (10, 11). This complexity can influence not only the decision to start biologic therapy but also adherence to the treatment regimen. IBD patients often overestimate the benefits while underestimating the risks associated with the infliximab drug (12). A further study in Ireland revealed that nearly 80% of the IBD patients from their sample were unaware of the safety profiles of biologics (13). These findings highlight a global challenge: insufficient patient knowledge and inaccurate perceptions may lead to suboptimal decision-making and treatment outcomes.

Although these studies offer valuable insights, more research is needed to elucidate the current understanding and attitude of patients toward this therapy. For this purpose, the Knowledge, Attitude, and Practice (KAP) studies are an instrumental approach to identify misconceptions, concerns, needs, and potential obstacles. These insights offer a critical foundation for optimizing public health initiatives, tailoring health education campaigns, and refining clinical interventions, which can potentially improve patient compliance (14).

Due to substantial variations in culture, economy, health literacy, healthcare systems, and government policies, KAP data are often highly specific to individual populations. In this sense, there is a shortage of KAP data concerning IBD among Chinese patients. To the best of our knowledge, only one study has evaluated their KAP toward general IBD notions (15), while none has specifically addressed treatment with biologics, especially among the Chinese population. This is, to our knowledge, the first study in China to comprehensively evaluate patients’ knowledge, attitudes, and decisional conflict regarding biologic therapy for IBD. By integrating decisional conflict assessment into a KAP framework, our work offers a novel, combined perspective that has not been addressed in previous literature. In China, where healthcare coverage for biologics may vary and patient–physician communication can be influenced by both cultural and systemic factors, understanding patients’ knowledge, attitudes, and decisional conflict regarding biologics is essential for guiding effective shared decision-making. The objective of this study was to assess the knowledge, attitudes, and decisional conflict related to biologic therapy among Chinese patients with IBD, with the goal of providing evidence to inform patient education and culturally tailored shared decision-making strategies.

## Materials and methods

### Study design and participants

This cross-sectional study was conducted at the author’s Hospital of Wenling City between July 2023 and January 2024 and focused on patients with IBD. The majority of patients were recruited through hospital-based patient groups and were diagnosed according to standard guidelines, including endoscopic, pathological, and surgical findings, and received long-term standard treatment. Exclusion criteria comprised patients with concomitant infectious enteritis such as bacterial dysentery, amoebic dysentery, and intestinal tuberculosis, as well as those with other intestinal diseases like ischemic enteritis and radiation enteritis. Additionally, patients with concurrent heart, lung, liver, kidney, or mental disorders, and individuals under the age of 18 were not included in the study. A total of 18 patients were excluded due to concomitant enteritis conditions, and 25 patients were excluded due to comorbid heart, lung, liver, kidney, or mental disorders. The study received ethical approval from the Ethics Committee of the author’s Hospital, and informed consent was obtained from all participants.

### Questionnaire

The questionnaire was conceptualized to capture three interrelated domains: knowledge, attitudes, and decisional conflict, based on the understanding that patient education (knowledge) influences perceptions (attitude), which in turn affects confidence in decision-making (decisional conflict). This conceptual alignment allowed for a coherent interpretation of results across domains. The design of this questionnaire was based on “*Expert consensus on biological therapy for IBD in China (2021)*” (16, 17) and previous literature (18). Modifications were made based on feedback from three specialists (one with expertise in small intestine diseases and two in IBD). Experts recommended the following: (1) listed the specific types of biologics; (2) investigated women’s fertility status; and (3) investigated patients’ awareness of the side effects of biologics. A preliminary survey showed good internal consistency with a Cronbach’s *α* of 0.874, based on 23 valid responses.

The final questionnaire was in Chinese and consisted of four sections: demographic data, knowledge dimension, attitude dimension, and decisional conflict scale (Supplementary materials). The demographic section gathered information on age, height, weight, gender, place of residence, educational attainment, average monthly family income, smoking and drinking habits, type and duration of IBD, history of intestinal surgery, current and past medications, current use of biologics, history of switching biologics, parental status, and continuation of biologic therapy during pregnancy or lactation. The knowledge dimension comprised six questions, with points awarded as follows: 2 points for “very familiar,” 1 point for “heard of,” and 0 points for “not sure,” applicable to questions 1–5. Question 6 was not scored, making the total possible score range from 0 to 10 points. The attitude dimension included six questions, scored using a five-point Likert scale. Possible scores ranged from 6 to 30. The decisional conflict scale consisted of 16 questions, also scored on a five-point Likert scale, with total scores ranging from 0 to 100. This score was calculated by summing all item scores, dividing by 16, and then multiplying by 25. Clinically significant decisional conflict was defined as a total score > 37.5 (19). According to Bloom’s cutoff, a well-established benchmark in educational environments (20), knowledge, or attitude of respondents is classified based on their scoring percentages. Scores below 60% of the total were deemed insufficient or lower level. Scores between 60 and 80% were considered moderate. Scores exceeding 80% were regarded as sufficient or higher level.

### Questionnaire distribution and quality control

The questionnaires were distributed through a combination of online and offline channels to ensure broad participation. The electronic questionnaire was imported into the Questionnaire Star platform^1^ and distributed through IBD doctor-patient communication WeChat groups, the gastroenterology outpatient clinic, and inpatient wards, effectively reaching participants familiar with digital tools. For electronic distribution, researchers introduced the purpose, content, and significance of the study before the survey. To ensure data completeness, electronic questionnaires could only be submitted after all items were filled out, and each IP address was allowed to answer the survey only once to avoid duplicate submissions. In contrast, the paper questionnaires were targeted at inpatients who were unable to use online scanning methods, ensuring inclusivity and comprehensive participation. For paper distribution, researchers also explained the purpose and content of the study. Trained staff distributed the questionnaires in person, and participants completed them voluntarily. The completed questionnaires were collected on-site.

The researchers ensured the integrity and internal consistency of the paper and electronic questionnaires, and incomplete or logically contradictory answers were considered invalid. This dual-channel distribution model (online and offline) was designed to maximize accessibility, minimize selection bias, and ensure the representativeness of the sample by including both digitally literate patients and those without access to online tools.

### Sample size calculation

A single population proportion formula, *n* = [(Z_α/2_)^2^*P(1-P)]/d^2^, was used to calculate the sample size. Since there were no KAP studies on inflammatory bowel disease in the Chinese population, the sample size for this study was calculated based on an expected proportion of patients’ understanding of biologic mechanisms at 50%, with a confidence level of 95% and a margin of error of 5%, requiring a sample size of 384 individuals. Considering a 10% dropout rate, the adjusted sample size required was 426 individuals. This calculation ensured sufficient statistical power to detect meaningful associations between patient characteristics and their knowledge, attitudes, and decisional conflict regarding biologics.

### Statistical analysis

The statistical analysis was structured to first describe the sample characteristics, then explore bivariate associations, and finally identify independent predictors through multivariate modeling. This hierarchical approach allowed for both descriptive insights and hypothesis-driven testing. Statistical analysis was performed using STATA 18.0 (Stata Corporation, College Station, TX, United States). Initially, continuous variables were tested for normality. Those that were normally distributed were presented as mean ± standard deviation (SD) and comparisons between groups were made using the t-test and one-way analysis of variance (ANOVA). Non-normally distributed variables were reported as median (range) and compared using the Mann–Whitney U test or the Kruskal-Wallis analysis of variance. Categorical variables were presented as n (%). The relationships between the knowledge and attitude dimensions were assessed using Spearman correlation analysis. Variables with *p* < 0.05 in the univariate logistic regression analysis were selected for the multivariate regression analysis. Multivariate logistic regression analysis was utilized with adequate knowledge and higher attitude as the dependent variables. A two-sided *p* < 0.05 was considered statistically significant. The alpha value of 0.05 was selected based on conventional statistical standards in clinical research. For multiple comparisons, we applied the Bonferroni correction. Specifically, since three main multivariate analyses were conducted, the corrected significance level was set at 0.0167.

This methodology was chosen over alternative approaches because the KAP framework allows for a structured, validated, and population-specific assessment of patient knowledge, attitudes, and decisional conflict. This is particularly important in the Chinese IBD context, where cultural and healthcare system factors influence treatment perceptions, and where comparable KAP data are lacking. The cross-sectional design combined with a targeted questionnaire enabled efficient, large-scale data collection and facilitated direct comparison with existing international studies.

## Results

### Characteristics of the participants

A total of 456 questionnaires were collected. After excluding 5 cases due to incomplete completion of the KAP section, 37 cases with illogical responses, and 6 questionnaires completed in less than 120 s, 405 valid questionnaires remained for analysis. Among the participants, 45.90% were aged 30–49, and 64.00% were male (Table 1). Among the medications previously or currently used by participants, the most common was mesalazine (87.2%), followed by steroids (26.4%) and azathioprine (21.0%). Traditional Chinese Medicine was used by 16.8%, while Sulfasalazine (4.2%) and Methotrexate (2.0%) were the least common. With respect to the biologics currently in use by the participants, adalimumab was the most commonly used (26.7%), followed by infliximab (25.9%) and vedolizumab (20.7%). Notably, 10.9% of participants were not using any biologics, and tofacitinib was not used by any participants.

**Table 1:** Basic characteristics.

Variables	N = 405 (%)	Knowledge		Attitude	
		Mean (± SD)	*p*	Mean (± SD)	*p*
Total Score	405(100.00)	6.03 (2.98)		24.36 (3.32)	
Age, year			<0.001		0.093
18–29	112(27.6)	6.61 (2.65)		24.62 (3.13)	
30–49	186(45.9)	6.47 (2.77)		24.62 (3.47)	
≥50	107(26.5)	4.67 (3.24)		23.62 (3.16)	
BMI, kg/m^2^			0.932		0.580
<18.5	75(18.5)	6.17 (2.88)		24.67 (3.18)	
18.5(inclusive)-24	254(62.7)	5.96 (3.04)		24.22 (3.36)	
24(inclusive)-28	63(15.6)	6.19 (2.92)		24.68 (3.17)	
≥28	13(3.2)	5.85 (2.91)		23.62 (4.03)	
Gender			0.126		0.809
Male	259(64.0)	6.20 (2.93)		24.41 (3.13)	
Female	146(36.0)	5.73 (3.05)		24.27 (3.65)	
Place of Residence			0.377		0.034
Rural	217(53.6)	6.20 (2.79)		24.71 (3.28)	
Urban	188(46.4)	5.84 (3.17)		23.95 (3.33)	
Education			<0.001		0.001
Primary School and below	32(7.9)	3.22 (3.18)		23.06 (3.77)	
Secondary School/Technical School	158(39.0)	5.79 (2.96)		23.68 (3.21)	
College and above	215(53.1)	6.63 (2.69)		25.05 (3.18)	
Average Household monthly Income, RMB			<0.001		<0.001
<5,000	132(32.6)	5.24 (3.05)		23.45 (3.20)	
5,000-10,000	158(39.0)	6.06 (3.04)		24.39 (3.29)	
10,000-20,000	85(21.0)	6.76 (2.58)		25.52 (3.24)	
>20,000	30(7.4)	7.30 (2.44)		24.90 (3.24)	
Smoking			0.041		0.024
Yes	34(8.4)	4.94 (3.29)		23.15 (3.12)	
No	371(91.6)	6.13 (2.93)		24.47 (3.32)	
Drinking			0.212		0.025
Yes	21(5.2)	5.00 (3.70)		22.81 (2.71)	
No	384(94.8)	6.09 (2.93)		24.44 (3.33)	
Type of IBD			<0.001		<0.001
Crohn’s Disease	282(69.6)	6.68 (2.79)		24.94 (3.05)	
Ulcerative Colitis	118(29.1)	4.59 (2.89)		23.10 (3.50)	
Not Yet Specified	5(1.2)	3.40 (1.82)		21.00 (4.36)	
Duration of IBD			0.163		0.413
<5 years	229(56.5)	6.20 (2.96)		24.48 (3.28)	
5 years and above	176(43.5)	5.82 (2.99)		24.20 (3.38)	
Intestinal surgery			0.002		0.318
Yes	82(20.2)	6.95 (2.63)		24.72 (2.89)	
No	323(79.8)	5.80 (3.02)		24.27 (3.42)	
Changed biologics			<0.001		<0.001
Changed	137(33.8)	6.88 (2.45)		25.15 (3.15)	
Not changed	221(54.6)	6.12 (2.94)		24.60 (2.90)	
Never using biologics	47(11.6)	3.15 (2.83)		20.89 (3.59)	
Fertility*			0.005		0.133
Yes	98(24.2)	5.18 (3.21)		23.90 (3.60)	
No	48(11.9)	6.83 (2.37)		25.04 (3.66)	
Continue to use biologics during pregnancy or lactation, either in the past or present*			0.209		0.641
Yes	15(3.7)	6.40 (2.92)		25.00 (3.05)	
No	131(32.3)	5.65 (3.07)		24.19 (3.71)	
Decisional Conflict Score			<0.001		<0.001
≤37.5	345(85.2)	6.53 (2.76)		25.04 (2.96)	
>37.5	60(14.8)	3.17 (2.52)		20.45 (2.51)	

*Among the female population; “fertility” refers to whether the participant has given birth to children.

### Knowledge, attitude, and decisional conflict

The mean scores of knowledge and attitude were 6.03 ± 2.98 (possible range: 0–11) and 24.36 ± 3.32 (possible range: 8–40). Among them, 60 (14.8%) patients exhibited significant decisional conflict. Overall, younger age, higher education level, higher household income, diagnosis of CD, prior use of biologics, and lower decisional conflict were consistently associated with higher knowledge and higher attitude scores toward biologic therapy. Detailed subgroup analyses are presented in Table 1. Participants aged 18–29 and 30–49 had higher knowledge scores compared to those aged ≥ 50 years (*p* < 0.001). Rural residents had higher attitude scores (*p* = 0.034). Higher education levels positively impacted both knowledge and attitude scores (*p* < 0.001, *p* = 0.001). Income levels influenced both domains, with higher incomes linked to higher scores (*p* < 0.001). Non-smokers and non-drinkers showed higher attitude scores, and non-smokers also had higher knowledge scores (*p* = 0.041, *p* = 0.024, and *p* = 0.025, respectively). Participants with CD had higher knowledge and attitude scores than those with other IBD conditions (*p* < 0.001). Changing biologics positively influenced knowledge and attitude (both *p* < 0.001). Patients with lower decisional conflict scores showed higher knowledge and attitude scores (*p* < 0.001; Table 1).

The majority of respondents (60.2%) were very familiar with biologics as a treatment option for IBD, while 32.8% had heard of them, and a small percentage (6.9%) were not sure. However, familiarity with the risks associated with biologics, such as increased infection and tumor risk, was low, with only 25.2% very familiar and 31.4% unsure. Additionally, only 1.5% were familiar with Tofacitinib, highlighting a significant gap in knowledge about specific biologics (Supplementary Table 1; Table 2).

**Table 2:** Univariate and multivariate analysis of knowledge.

Variables	Univariate analysis	*p*	Multivariate analysis	*p*
	OR (95%CI)		OR (95%CI)	
Age, year				
18–29				
30–49	1.04(0.63,1.70)	0.867	1.26 (0.68,2.32)	0.462
≥50	0.31(0.17,0.53)	**<0.001**	0.46 (0.22,0.97)	0.040
BMI, kg/m^2^				
<18.5				
18.5(inclusive)-24	0.71(0.41,1.20)	0.203		
24(inclusive)-28	0.62(0.31,1.23)	0.176		
≥28	0.62(0.19,2.10)	0.429		
Gender				
Male				
Female	0.65(0.43,0.98)	**0.042**	0.71 (0.42,1.19)	0.194
Place of Residence				
Rural				
Urban	0.85(0.57,1.26)	0.410		
Education				
Primary School and below				
Secondary School/Technical School	4.27(1.83,11.21)	**0.001**	2.79 (0.92,8.41)	0.069
College and above	6.95(3.01,18.1)	**<0.001**	3.04 (0.94,9.86)	0.064
Average Household monthly Income, RMB				
<5,000				
5,000-10,000	1.53(0.96,2.45)	**0.072**	1.28 (0.72,2.26)	0.401
10,000-20,000	2.71(1.53,4.89)	**0.001**	1.94 (0.92,4.09)	0.080
>20,000	3.1(1.33,7.89)	**0.011**	2.43 (0.84,7.00)	0.100
Smoking				
Yes				
No	1.85(0.91,3.80)	0.090	2.02 (0.84,4.84)	0.117
Drinking				
Yes				
No	1.27(0.52,3.09)	0.591		
Type of IBD				
Crohn’s Disease				
Ulcerative Colitis	0.28(0.18,0.44)	**<0.001**	0.53 (0.31,0.93)	0.026
Not Yet Specified	/	0.980	/	0.984
Duration of IBD				
<5 years				
5 years and above	2.00(1.00,4.04)	0.298		
Intestinal surgery				
Yes				
No	0.81(0.54,1.21)	**0.005**	0.54 (0.27,1.06)	0.072
Changed biologics				
Changed				
Not changed	0.70(0.45,1.10)	0.128	0.62 (0.37,1.06)	0.079
Never using biologics	0.08(0.03,0.18)	**<0.001**	0.16 (0.06,0.45)	0.001
Decisional Conflict Score				
≤37.5				
>37.5	0.09(0.04,0.20)	**<0.001**	0.13 (0.06,0.29)	**<0.001**

Bold values indicate *P* < 0.05.

Among the participants, 81.5% agreed or strongly agreed that biologics would improve their condition, and 88.4% were willing to accept biologic therapy if recommended by their doctor. However, concerns about side effects were noted, with 13.1% expressing resistance or hesitancy toward these treatments. Additionally, financial considerations played a critical role, with only 68.1% feeling confident in their ability to afford biologic therapy, even with insurance coverage (Supplementary Table 2).

In terms of decision conflict, the majority of participants (23% strongly agree and 50.4% agree) reported having a clear understanding of their treatment options. Furthermore, 89.9% (27.4% strongly agree and 62.5% agree) felt no external pressure in making their decision, and 84.7% (22.2% strongly agree and 62.5% agree) expressed satisfaction with their choice (Supplementary Table 3).

In an additional subgroup analysis, patients who reported using Traditional Chinese Medicine (TCM) had lower attitude scores compared to those who did not use TCM (23.19 ± 3.49 vs. 24.59 ± 3.24, *p* = 0.001). No statistically significant differences were observed for knowledge scores (5.54 ± 3.15 vs. 6.13 ± 2.93, *p* = 0.139) or decisional conflict (26.93 ± 13.33 vs. 23.87 ± 13.32, *p* = 0.085; Supplementary Table 4).

### Correlation and multivariate analysis

Spearman correlation analysis showed that knowledge scores were positively correlated with attitude scores (*r* = 0.554, *p* < 0.001). In the multivariate analysis for knowledge, age ≥50 years (OR = 0.46, 95% CI [0.22–0.97], *p* = 0.04), UC (OR = 0.53, 95% CI [0.31–0.93], *p* = 0.026), never having used biologics (OR = 0.16, 95% CI [0.06–0.45], *p* = 0.001), and clinically significant decisional conflict (OR = 0.13, 95% CI [0.06–0.29], *p* < 0.001) were each associated with lower odds of having adequate knowledge.

In the multivariate analysis for attitude, higher knowledge score was associated with higher attitude (OR = 1.32, 95% CI [1.19–1.46], *p* < 0.001). Monthly household income of 10,000–20,000 RMB was also positively associated with higher attitude (OR = 2.20, 95% CI [1.01–4.78], *p* = 0.048). In contrast, never having used biologics (OR = 0.16, 95% CI [0.06–0.46], *p* = 0.001) and clinically significant decisional conflict (OR = 0.08, 95% CI [0.03–0.23], p < 0.001) were associated with lower odds of higher attitude (Table 3).

**Table 3:** Univariate and multivariate analysis of attitude.

Variables	Univariate analysis	*p*	Multivariate analysis	*p*
	OR (95%CI)		OR (95%CI)	
Knowledge score	1.47(1.35,1.60)	**<0.001**	1.32 (1.19,1.46)	**<0.001**
Age, year				
18–29				
30–49	1.00(0.61,1.63)	0.993	1.47 (0.79,2.73)	0.225
≥50	0.61(0.35,1.05)	0.074	2.01 (0.90,4.46)	0.088
BMI, kg/m^2^				
<18.5				
18.5(inclusive)-24	0.82(0.48,1.38)	0.459		
24(inclusive)-28	1.19(0.59,2.42)	0.625		
≥28	0.95(0.29,3.42)	0.938		
Gender				
Male				
Female	0.92(0.61,1.39)	0.678		
Place of Residence				
Rural				
Urban	0.80(0.54,1.20)	0.284		
Education				
Primary School and below				
Secondary School/Technical School	1.39(0.65,3.02)	0.402	0.59 (0.20,1.74)	0.337
College and above	2.84(1.34,6.14)	**0.007**	1.18 (0.37,3.80)	0.781
Average Household monthly Income, RMB				
<5,000				
5,000-10,000	1.65(1.03,2.63)	**0.037**	1.29 (0.71,2.33)	0.397
10,000-20,000	3.24(1.80,5.99)	**<0.001**	2.20 (1.01,4.78)	0.048
>20,000	2.12(0.94,5.06)	0.076	0.92 (0.33,2.61)	0.880
Smoking				
Yes				
No	1.79(0.88,3.67)	0.104		
Drinking				
Yes				
No	2.10(0.87,5.27)	0.101		
Type of IBD				
Crohn’s Disease				
Ulcerative Colitis	0.31(0.20,0.48)	**<0.001**	0.68 (0.38,1.21)	0.189
Not Yet Specified	0.30(0.04,1.86)	0.195	0.74 (0.06,8.90)	0.812
Duration of IBD				
<5 years				
5 years and above	0.96(0.64,1.43)	0.832		
Intestinal surgery				
Yes				
No	0.69(0.41,1.15)	0.159		
Changed biologics				
Changed				
Not changed	0.64(0.40,1.01)	0.057	0.71 (0.41,1.24)	0.226
Never using biologics	0.07(0.03,0.15)	**<0.001**	0.16 (0.06,0.46)	0.001
Decisional Conflict Score				
≤7.5				
>37.5	0.04(0.02,0.10)	**<0.001**	0.08 (0.03,0.23)	**<0.001**

Bold values indicate *P* < 0.05.

## Discussion

This study suggested that patients generally had moderate knowledge but positive attitude toward biologics. Older age, having UC, lack of prior biologic use, and decisional conflict were found to be associated with lower knowledge and lower attitude. Our findings highlight the importance of improving patient education and support systems to reduce decisional conflict and help patients become more confident and informed in managing their disease.

Numerous studies have previously highlighted misconceptions and a general lack of understanding among IBD patients concerning their condition, with ongoing issues in countries such as New Zealand (21), Canada (22), Israel (23), Poland (24) and South Korea (25). This general picture suggests that there is significant room for improvement in patient education, particularly in understanding the efficacy and safety of treatments. A similar conclusion may be extracted from the moderate knowledge levels of our population sample, which evidenced several gaps in their understand of biologics as a treatment for IBD. On the contrary, a previous study on the KAP of patients living with IBD in Wenzhou, China, evidenced good general knowledge about IBD (15). Despite they did not specifically focus on biologics therapy, which could explain the knowledge differences to our results, they found a high rate of correct answers about biologics specifically, which could denote higher knowledge about biologics in their sample population than in ours. These discrepancies may be due to local differences between these Chinese regions in demographic and clinical terms.

According to our multivariate analysis, knowledge levels were lower among elderly, patients with CD (versus those with UC), and biologic users. Addressing the present age differences requires a multifaceted approach that considers healthcare engagement, socioeconomic factors, communication preferences, and psychological barriers. On the other hand, the disparity in knowledge levels between patients with different types of IBD is also an aspect to be considered, as it underlines the need for disease-specific educational programs that address the unique aspects and concerns of each condition. Noteworthy, since the use of biologics was associated with higher knowledge, integrating comprehensive educational discussions into treatment planning sessions, especially when introducing the idea of biologics, could be beneficial. This strategy could also help those who have not yet used biologics but are potential candidates for such treatments. Different independent predictors for knowledge about IBD among the Chinese were found in the Shao et al. study (15), where age and education were independent predictors of knowledge. In particular, middle school, high school, and higher education levels were associated with better knowledge. These differences may result from the unique socio-cultural contexts of different patient populations.

Knowledge, income, and use of biologics were determined as independent predictors of attitude. In line with our findings, Shao et al. (15) also observed a positive correlation between knowledge and attitude scores. These results denote that better-informed patients tend to have higher attitude toward their disease management. Given that effective KAP related to IBDs are linked to improved outcomes (25), enhancing these aspects can lead to better patient results, especially since self-management plays a central role in improving quality of life in IBD (16). Conversely, the work by Shao et al. (15) did not highlight income as an independent predictor. These differences underscore the potential impact of cultural and socioeconomic differences between the populations studied. In our study, patients who had never used biologics exhibited very strong negative attitude toward their available treatment options. This might reflect a lack of familiarity or understanding of the potential benefits of these treatments, highlighting the importance of educating new patients about all available treatment modalities. In China, the high cost of biologics remains a substantial barrier to their use, despite the inclusion of some biologic agents in the National Reimbursement Drug List in recent years. Reimbursement coverage varies across provinces, and out-of-pocket expenses can still be considerable for many patients. This financial burden may limit patients’ access to detailed information about biologic therapies, as physicians might be less inclined to discuss treatment options that they perceive to be unaffordable for the patient. Consequently, economic constraints may indirectly influence patient knowledge by narrowing the scope of treatment discussions, underscoring the importance of addressing affordability alongside educational interventions (7, 11, 17).

The findings that a majority of patients felt supported in their decisions, were not feeling pressured by others, and were confident in their treatment choices suggested a positive environment for patient autonomy and empowerment. However, fewer found making decisions easy or felt fully informed, indicating that further support and clarification could be beneficial. The significant link between decisional conflict and knowledge and attitude points to the importance of education and clear and effective communication in healthcare settings. Ensuring that patients understand their treatment options fully can reduce uncertainty and foster more positive attitude toward their chosen treatments.

This study has some limitations. It was conducted at a single center, which may limit the generalizability of the findings to the broader IBD patient population. Additionally, patients with comorbid heart, lung, liver, kidney, or mental disorders were excluded from the study, which may further limit the generalizability of the findings to the overall IBD population. The inherent biases of a single-center study, including demographic and socio-economic homogeneity, could influence the generalizability of the results. KAP surveys capture the state of a specific population at a particular moment. Consequently, conducting studies in other populations and at different times is essential to assess the current KAP status in China and the impact of educational initiatives. Additionally, all KAP surveys are vulnerable to social desirability bias, where participants might be inclined to give responses they believe are expected rather than their true opinions. Lastly, the sample size, while adequate for statistical analysis, is relatively small and may not fully capture the diversity of the IBD population, especially in terms of varying disease severities and the spectrum of biologic therapy used. Due to the study design, the response rate could not be calculated, as the number of individuals who were invited to participate but did not respond was not recorded.

## Conclusion

IBD patients demonstrated moderate knowledge and higher attitude toward biologics. Healthcare providers should tailor information and support to meet the specific needs of different patient groups, continually reassessing and reinforcing knowledge and attitude about treatment. Addressing factors such as age, disease type, prior biologic use, and decisional conflict can help identify patients who may benefit most from targeted interventions. By improving understanding and reducing decisional conflict, healthcare professionals can empower patients to make more informed treatment decisions. This approach will not only optimize biologic therapy use but also enhance patient adherence, improve long-term treatment outcomes, and ultimately, improve the quality of life for patients managing IBD.

## Data Availability

The original contributions presented in the study are included in the article/Supplementary material, further inquiries can be directed to the corresponding author.

## Footnotes

^1^ https://www.wjx.cn/
