# Supplementary material for: Knowledge, and attitude, and decisional conflict regarding biologics among patients with inflammatory bowel disease: a cross-sectional study
Source: Front Med (Lausanne). 2025 Oct 9;12:1604851. doi: 10.3389/fmed.2025.1604851 (PMC12546363; doi:10.3389/fmed.2025.1604851)
Supplement: Supplementary file 1 [file Table_1.DOCX]

**Supplementary Table 1. Distribution of Knowledge Scores**

| **Knowledge** | N（%） | | |
| --- | --- | --- | --- |
|  | Very familiar | Heard of it | Not sure |
| Are you aware that biologics are a treatment option for inflammatory bowel disease (IBD)? | 244(60.2%) | 133(32.8%) | 28(6.9%) |
| Are you aware that biologics are recommended for Crohn's disease (CD) patients at high risk of postoperative recurrence? | 143(35.3%) | 146(36%) | 116(28.6%) |
| Are you aware that biologics are recommended as the first-line treatment for moderate to severe ulcerative colitis patients? | 159(39.3%) | 166(41%) | 80(19.8%) |
| Are you aware that using biologics can reduce disease activity, disease recurrence rate, and the need for steroids? | 188(46.4%) | 150(37%) | 67(16.5%) |
| Are you aware that biologic therapy may also carry side effects such as increased risk of infection and potential tumor risk? | 102(25.2%) | 176(43.5%) | 127(31.4%) |

**Supplementary Table 2. Distribution of Attitude Scores**

| **Attitude** | Strongly Agree | Agree | Neutral | Disagree | Strongly Disagree |
| --- | --- | --- | --- | --- | --- |
| I believe that treatment with biologics will help improve my condition. | 174(43%) | 156(38.5%) | 74(18.3%) | 0 (0%) | 1(0.2%) |
| If my doctor recommends treatment with biologics, I am willing to accept it. | 154(38%) | 204(50.4%) | 42(10.4%) | 4(1%) | 1(0.2%) |
| I believe that biologics will reduce my need for steroids / I have already stopped using steroids. | 147(36.3%) | 170(42%) | 80(19.8%) | 8(2%) | 0 (0%) |
| I believe that hospitals can regularly educate patients about IBD and treatment with biologics. | 224(55.3%) | 155(38.3%) | 25(6.2%) | 0 (0%) | 1(0.2%) |
| I believe that biologics have significant side effects, and I would be very resistant to treatment or unable to persist. | 23(5.7%) | 30(7.4%) | 150(37%) | 164(40.5%) | 38(9.4%) |
| I believe that all biologics are covered by medical insurance / I have the financial ability to undergo treatment with biologics. | 103(25.4%) | 173(42.7%) | 101(24.9%) | 23(5.7%) | 5(1.2%) |

**Supplementary Table 3. Distribution of Decisional Conflict Scale Scores**

| **Decisional Conflict Scale** | Strongly agree | Agree | Neutral | Disagree | Strongly disagree |
| --- | --- | --- | --- | --- | --- |
| I know what my options are (to undergo biologic therapy or not). | 93(23%) | 204(50.4%) | 101(24.9%) | 5(1.2%) | 2(0.5%) |
| I know the benefits of undergoing (or not) biologic therapy. | 98(24.2%) | 239(59%) | 64(15.8%) | 3(0.7%) | 1(0.2%) |
| I know the potential risks and side effects of undergoing (or not) biologic therapy. | 88(21.7%) | 242(59.8%) | 71(17.5%) | 3(0.7%) | 1(0.2%) |
| I am clear about which benefits of treatment with treatment with biologics are most important to me. | 92(22.7%) | 211(52.1%) | 86(21.2%) | 12(3%) | 4(1%) |
| I am clear about which risks and side effects of biologic therapy are most concerning to me. | 101(24.9%) | 255(63%) | 47(11.6%) | 2(0.5%) | 0 (0%) |
| I am clear about what is more important to me (the benefits of biologic therapy or the risks and side effects). | 101(24.9%) | 257(63.5%) | 45(11.1%) | 2(0.5%) | 0 (0%) |
| I have received enough support from others to make a choice (to undergo biologic therapy or not). | 109(26.9%) | 251(62%) | 45(11.1%) | 0 (0%) | 0 (0%) |
| I do not feel pressured by others when making my decision. | 111(27.4%) | 253(62.5%) | 40(9.9%) | 1(0.2%) | 0 (0%) |
| I have received enough advice to make this decision (to undergo biologic therapy or not). | 92(22.7%) | 211(52.1%) | 100(24.7%) | 2(0.5%) | 0 (0%) |
| I am clear about which option is best for me (to undergo biologic therapy or not). | 80(19.8%) | 204(50.4%) | 119(29.4%) | 2(0.5%) | 0 (0%) |
| I know what to choose (i.e., I am confident in my treatment choice). | 88(21.7%) | 243(60%) | 72(17.8%) | 2(0.5%) | 0 (0%) |
| Making this decision (to undergo biologic therapy or not) is easy for me. | 75(18.5%) | 207(51.1%) | 118(29.1%) | 4(1%) | 1(0.2%) |
| I feel that I am making an informed decision (fully understanding the situation). | 84(20.7%) | 232(57.3%) | 87(21.5%) | 2(0.5%) | 0 (0%) |
| My decision reflects what is important to me. | 79(19.5%) | 228(56.3%) | 91(22.5%) | 5(1.2%) | 2(0.5%) |
| I hope to stick to my decision. | 101(24.9%) | 258(63.7%) | 44(10.9%) | 2(0.5%) | 0 (0%) |
| I am satisfied with my decision. | 90(22.2%) | 253(62.5%) | 60(14.8%) | 1(0.2%) | 1(0.2%) |

**Supplementary Table 4. Comparison of knowledge, attitude, and decisional conflict scores between patients using Traditional Chinese Medicine (TCM) and those not using TCM.**

| **Variable** | Non-TCM Users (n=337) | TCM Users (n=68) | P |
| --- | --- | --- | --- |
| Knowledge, mean (± SD) | 6.13 (2.93) | 5.54 (3.15) | 0.139 |
| Attitude, mean (± SD) | 24.59 (3.24) | 23.19 (3.49) | 0.001 |
| Decisional conflict, mean (± SD) | 23.87 (13.32) | 26.93 (13.33) | 0.085 |
